# P2R Inhibitors Prevent Antibody-Mediated Complement Activation in an Animal Model of Neuromyelitis Optica: P2R Inhibitors Prevent Autoantibody Injury

**DOI:** 10.1007/s13311-022-01269-w

**Published:** 2022-07-12

**Authors:** Sudhakar Reddy Kalluri, Rajneesh Srivastava, Selin Kenet, Goutam K. Tanti, Klaus Dornmair, Jeffrey L. Bennett, Thomas Misgeld, Bernhard Hemmer, Matthias T. Wyss, Marina Herwerth

**Affiliations:** 1 Department of Neurology, Klinikum Rechts Der Isar, Technical University of Munich, Munich, Germany; 2 Institute of Neuronal Cell Biology, Technical University of Munich, Munich, Germany; 3 Graduate School of Systemic Neurosciences, Ludwig-Maximilians-Universität, Munich, Germany; 4 Institute of Clinical Neuroimmunology, University Hospital and Biomedical Center, LMU Munich, Munich, Germany; 5 Departments of Neurology and Ophthalmology, Programs in Neuroscience and Immunology, University of Colorado School of Medicine, Colorado, USA; 6 Munich Cluster of Systems Neurology (SyNergy), Munich, Germany; 7 German Center for Neurodegenerative Diseases (DZNE), Munich, Germany; 8 Institute of Pharmacology and Toxicology, University of Zurich, Zurich, Switzerland; 9 Neuroscience Center Zurich, University Zurich and ETH Zurich, Zurich, Switzerland

**Keywords:** Aquaporin-4, Neuromyelitis optica, Astrocytes, Purinergic receptor blockers, Two-photon imaging, Suramin

## Abstract

Purinergic 2 receptors (P2Rs) contribute to disease-related immune cell signaling and are upregulated in various pathological settings, including neuroinflammation. P2R inhibitors have been used to treat inflammatory diseases and can protect against complement-mediated cell injury. However, the mechanisms behind these anti-inflammatory properties of P2R inhibitors are not well understood, and their potential in CNS autoimmunity is underexplored. Here, we tested the effects of P2R inhibitors on glial toxicity in a mouse model of neuromyelitis optica spectrum disorder (NMOSD). NMOSD is a destructive CNS autoimmune disorder, in which autoantibodies against astrocytic surface antigen Aquaporin 4 (AQP4) mediate complement-dependent loss of astrocytes. Using two-photon microscopy in vivo, we found that various classes of P2R inhibitors prevented AQP4-IgG/complement-dependent astrocyte death. In vitro, these drugs inhibited the binding of AQP4-IgG or MOG-IgG to their antigen in a dose-dependent manner. Size-exclusion chromatography and circular dichroism spectroscopy revealed a partial unfolding of antibodies in the presence of various P2R inhibitors, suggesting a shared interference with IgG antibodies leading to their conformational change. Our study demonstrates that P2R inhibitors can disrupt complement activation by direct interaction with IgG. This mechanism is likely to influence the role of P2R inhibitors in autoimmune disease models and their therapeutic impact in human disease.

**Supplementary Information:**

The online version contains supplementary material available at 10.1007/s13311-022-01269-w.

## Introduction

The importance of purinergic signaling in neuroinflammation is an evolving field that has grown rapidly over the last two decades [1, 2]. Purinergic 2 receptors (P2Rs) are known to be linked to secretion of the key cytokine interleukin 1β [3], to be upregulated after brain and spinal cord injury [4–7] and to contribute to neuronal-glial interaction [8]. For instance, ATP via P2R mediates calcium waves in astrocytes and in reciprocal neuron-glia signaling [9] and contributes to sustained reactive astrogliosis [10, 11]. Of the different P2R subtypes, P2X and P2Y receptors are expressed on all types of nervous system cells [10], including astrocytes. Astrocytes are known to express P2X1-5 and P2X7 receptors [12, 13], as well as P2Y1-2, P2Y4, P2Y6, P2Y12, and P2Y14 receptors [14]. Moreover, in inflammatory conditions, purinergic receptors are enriched on different immune cells, mediating important functions such as cell proliferation, chemoattraction, and release of inflammatory cytokines [15].

Due to the important role of purinergic signaling in inflammatory processes [2, 7, 16], purinergic inhibitors have been studied for the treatment of various inflammatory diseases, where they have shown cell-protective effects against complement-mediated cell injury [17–21]. Indeed, purinergic inhibitors, such as suramin, have a broad spectrum of clinical applications, from parasitic and viral diseases to cancer and autism [16]. Moreover, it has been recently shown that suramin is a potent inhibitor of the SARS-CoV-2 RNA polymerase [22]. However, the mechanisms behind their anti-inflammatory properties are not well understood. The effects of purinergic inhibitors on CNS autoimmune conditions, such as neuromyelitis optica spectrum disorder (NMOSD), have not been investigated.

NMOSD is a destructive autoimmune disease of the central nervous system (CNS). Patients suffer from severe relapses affecting especially the optic nerve, spinal cord, and brainstem [23]. The autoantigen in the majority (> 80%) of NMOSD patients [24] is the water channel aquaporin 4 (AQP4), which in the CNS is only expressed by astrocytes and ependymal cells [25, 26]. A key mechanism of injury in NMOSD pathology is believed to be the binding of AQP4 antibodies to astrocytes, causing a complement-mediated astrocyte necrosis, immune cell infiltration [27, 28], and tissue edema. Finally, NMOSD lesions show demyelination and reduction in axon density [25, 26]. Another autoimmune disease of CNS that also presents with myelitis and optic neuritis is the MOG antibody-associated disorder (MOGAD) [29–31]. MOGAD patients have antibodies against myelin-oligodendrocytic glycoprotein (MOG-IgG). However, the pathology of MOGAD seems to be distinct from AQP4-IgG seropositive NMOSD [31].

In order to explore whether blocking purinergic signaling could be beneficial in NMOSD, we tested P2R inhibitors in an NMOSD mouse model that we have previously established [32, 33]. In this model, two-photon in vivo imaging of the spinal cord reveals the dynamics of AQP4-IgG/complement-mediated astrocyte death and allows following NMOSD lesion formation over time. Surprisingly, the P2R inhibitors suramin, NF449, and PPADS prevented AQP4-IgG-mediated complement deposition and astrocyte death in the spinal cord. Using a cell-based antibody-binding assay, size-exclusion chromatography (SEC), and circular dichroism (CD) spectroscopy of IgG molecules, we demonstrate that this is likely due to a concentration-dependent partial unfolding of IgG by P2R inhibitors, thus disrupting complement activation, needed for AQP4-IgG-mediated astrocyte death.

## Methods

### Patient Sera, Antibodies, and Complement Source

AQP4-IgG- and MOG-IgG-positive samples were collected from patients treated in the Department of Neurology, Klinikum rechts der Isar, Technical University of Munich, Germany. Samples were stored in the biobank of the Department of Neurology, which is part of the Joint Biobank Munich in the framework of the German Biobank Node. All cases fulfilled the Wingerchuk’s diagnostic criteria for NMOSD 2015 [23]. AQP4-IgG-positive NMOSD plasma was heat-inactivated for in vivo experiments. In some experiments, a human IgG_1_ recombinant antibody rAQP4^7−5–53^-IgG reconstructed from a clonally expanded NMOSD patient CSF plasma blast was used [34]. As anti-MOG-antibody, we used a humanized recombinant version (human IgG1- and κ-regions) [35, 36] of the mouse monoclonal anti-MOG antibody 8-18C5 (IgG1) [37]. Three different sera of healthy subjects, obtained from the blood bank of the Bavarian Red Cross, were pooled and served as complement source. All subjects gave written informed consent to the use of their blood samples for research purposes.

### Cell-Based Antibody Binding Assay

AQP4- and MOG-IgG binding was measured by using a cell-based flow cytometry assay. The human glioblastoma cell line, LN18, was used to stably overexpress human AQP4-M23 (301 aa; LN18-AQP4) and human full-length MOG (247 aa; LN18-MOG) individually as previously described [34, 38, 39]. Results are expressed as difference in median fluorescence intensity (ΔMFI) corrected for background binding to a cell line that was transduced with an empty vector pLenti6/V5 (LN18-CTR). To measure AQP4- or MOG-specific IgG either in the absence or in the presence of P2R inhibitors, we selected AQP4-positive or MOG-positive samples or used recombinant monoclonal antibodies (rAQP4^7−5–53^-IgG and r8-18C5-IgG) as primary antibodies. LN18-AQP4, LN18-MOG, or LN18-CTR cell lines were incubated with respective primary antibodies in individual U-shaped wells of a 96-well plate. Alexa-488 conjugated goat anti-human IgG H + L (Thermo Fisher Scientific #A-11013) was used as secondary antibody. In all flow cytometry–based measurements, both primary and secondary antibodies were diluted in the ratio of 1 to 100 in FACS buffer (2% FCS in PBS pH7.4), and for each staining step, cells were incubated for 30 min at 37 °C either in the absence or in the presence of P2R inhibitors. IgG-binding affinity was measured in two different conditions. In the first condition, cell lines were stained with (1) primary antibodies and continued with incubation (30 min) of (2) P2R inhibitors in different concentrations (1 µM, 10 µM, 100 µM, 500 µM, 1000 µM) and followed by (3) secondary antibodies. In the second condition, (1) P2R inhibitors were also incubated for 30 min at the same concentrations as above with cell lines, followed by (2) 30-min incubation with the primary antibodies and then (3) secondary antibodies. In both conditions, cells were washed twice after every staining step. Antibody binding strength in the absence or in the presence of P2R inhibitors on respective cell lines was quantified on a CytoFLEX S flow cytometer (Beckman Coulter, Brea, USA).

### IgG Purification, SDS PAGE, Size-Exclusion Chromatography

#### IgG Purification

Total IgGs from AQP4-IgG positive NMOSD patient sera purified by using Protein G GraviTrap columns (Cytiva # 28–9852-55) according to the manufacturer’s instructions. Fifty micrograms of NMO-IgGs was incubated without or with suramin (200 µM) in phosphate-buffered saline (1xPBS; pH 7.4) for 2 h. Afterwards, IgGs were analyzed in SDS PAGE or in SEC columns.

#### SDS PAGE Analysis

IgGs were loaded in equal amounts into a NuPAGE 4–12% Bis–Tris gel (Thermo Fisher Scientific no. NP0322BOX) to separate the proteins. The colloidal blue staining kit (Thermo Fisher Scientific no. LC6025) was used to visualize protein bands after electrophoresis according to the manufacturer’s instructions (SeeBlue™ Plus2 Pre-stained Protein Standard, Thermo Fisher Scientific #LC5925).

#### Size-Exclusion Chromatography Column

IgGs (50 µg of each for NMO-IgG, rAQP4^7−5–53^-IgG, or r818C5-IgG) were treated with or without suramin at different concentrations (100 µM and 1000 µM) in PBS (1 × , pH 7.4) and IgGs were loaded in native condition and analyzed using a SEC column (Superose 6 Increase 10/300 GL, GE no. 29–0915-96) on Äkta pure protein purification system (Cytiva). Absorption was measured at 280 nm as milli-Absorbance Units (mAU).

### Far-UV CD Spectroscopy

Circular dichroism (CD) spectra were measured with a Chirascan V100 CD spectrometer (Applied Photophysics Ltd., Leatherhead, Surrey, UK). For the measurements, a quartz cuvette having a light path length of 0.1 cm was used. Measurements were performed at 25 °C. For the far-UV measurements, the concentration of rAQP4^7−5–53^-IgG was 0.15 mg/ml (in PBS). Spectra in the 195 to 300 nm wavelength range (1 nm resolution) were acquired with a scan rate of 240 nm/min and a time constant of 0.25 s. The final spectra are the average of 5 scans for each condition. Presented data is background corrected.

### Animals

All mice were 2- to 5-month-old *Aldh1l1:*GFP mice, in which astrocytes are fluorescently labeled with green fluorescent protein (GFP), obtained from MMRRC (strain: Tg(Aldh1l1-EGFP)OFC789Gsat/Mmucd). Animal experiments were conducted in accordance with local regulations and were approved by the responsible regulatory agencies.

### Mouse NMOSD Model

#### Surgical Procedures

Laminectomy surgery was performed as previously described [32, 40, 41]. In brief, mice were anesthetized by an intraperitoneal injection of medetomidine 0.5 mg/kg, midazolam 5 mg/kg, and fentanyl 0.05 mg/kg. Anesthesia was reapplied as needed. After a double dorsal laminectomy over the L3 and L4 segments, mice were suspended using compact spinal cord clamps [42]. An imaging window free from dura was established using a bent hypodermic needle. The established window was superfused with artificial cerebrospinal fluid (aCSF containing in mM: 148.2 NaCL, 3.0 KCl, 0.8 Na_2_HPO_4_, 0.2 NaH_2_PO_4_, 1.4 CaCl_2_, and 0.8 MgCl_2_). To hold aCSF, a well around the opening was built using 2–3% agarose.

#### In Vivo Imaging

In vivo imaging of the lumbar spinal cord was performed as previously described [32, 40, 43]. Briefly, stacks were acquired using two-photon microscopes (Olympus FV1000 MPE or FVMPE-RS) tuned to 920 nm to elicit green fluorescent protein (GFP). The system was equipped with a 25 × /1.05 N.A. water immersion objective. Excitation and emission light was separated by a 690-nm short-pass dichroic mirror. Green fluorescence signal was detected by a gallium arsenide phosphide (GaAsP) photomultiplier tube equipped with a G filter (BA495-540). Time-lapse stacks were acquired at 10-min intervals for 4 h with the following parameters: 30–50 images (zoom 2.0; xy pixel size: 0.28 µm) with 1–2 µm z-steps.

Heat-inactivated AQP4-IgG-positive patient plasma (total IgG 150 µg/ml) together with 20% of healthy donor serum as a complement source was applied every 30 min for the first 2 h, afterwards, the solution was refreshed every 60 min. Under these experimental conditions, we have previously shown that phototoxicity and transgenic labeling do not significantly influence cellular health [32, 40, 41, 43].

### Pharmacology

NF449, A-438079, PPADS, and MRS2179 were obtained from Tocris Bioscience, suramin was obtained from Sigma Aldrich, and A-317491 was ordered from the Abcam laboratory. All purinergic inhibitors were dissolved in aCSF as a 30–70 mM stock solution and aliquoted accordingly.

### Immunohistochemistry and Confocal Imaging

Immediately after the experiment, mice were perfused transcardially with 4% paraformaldehyde (PFA) in 0.1 M of phosphate-buffered saline (1xPBS; in mM: 1.5 KH_2_PO_4_, 2.7 KCl, 8.1 Na_2_HPO_4_, and 137 NaCl), followed by an additional overnight fixation in PFA. On the next days, whole mounts of lumbar spinal cord were extracted and kept in well plates filled with 1xPBS for further staining procedures. For immunohistochemical analysis, spinal cords were put in a sucrose (30%) solution for further 12 to 24 h. Subsequently, 20-µm-thick cryosections were cut in a cryostat. Antibodies were diluted in 0.2% Triton X-100, 10% normal goat or donkey serum, and 1% bovine serum albumin in 1xPBS. C5b-9 (MAC) antibody (mouse monoclonal anti-human, Dako, #M0777) was used at a concentration of 1:200, followed by goat Alexa Fluor-647-conjugated anti-mouse IgG2a IgG (1:1000). C3 antibody (polyclonal rabbit anti-human, Dako, #A006302-2, 1:200) incubation was followed by donkey Alexa Fluor-647-conjugated anti-rabbit secondary antibody (1:1000). Sections were mounted in 4,6-diamidino-2-phenylindole (DAPI)-containing mounting medium. Samples were scanned with an inverted confocal microscope (Zeiss, LSM700) equipped with 20 × /0.8 N.A. and 60 × /1.42 N.A. oil-immersion objectives. For quantification, one randomly selected standardized region of interest (ROI; depth from pial surface x length parallel to pia: 40 µm × 200 µm) in the dorsal spinal cord was imaged per animal in the laminectomy (NMO) area. Microscope settings were kept identical when acquiring images of different conditions.

### Image Processing/Representation

Images were processed using the open-source image analysis software Fiji [44] and the image-processing software, Adobe Creative Suite (CS6). The quantitative analysis of MAC and C3 stainings was performed by a scorer blinded for the experimental condition. Mean fluorescence intensity was averaged for each area, and local mean background intensity (determined in the same image) was subtracted. Numerical datasets were processed with Excel (Microsoft Corporation, Redmond, WA). In non-quantitative panels, gamma was adjusted non-linearly to enhance visibility of low-intensity objects.

### Data Analysis

Results are presented as mean ± SEM. Statistical significance was analyzed with the GraphPad Prism 7 software using a nonparametric *t*-test followed by Mann–Whitney test for comparing two groups and nonparametric ANOVA followed by Kruskal–Wallis test for comparing more than two groups. For CD spectroscopy data, a paired parametric *t*-test was used. *P* values < 0.05 were considered to be significant and indicated by “*”, *p* values < 0.01 by “**”, < 0.001 by “***”, < 0.0001 by “****”.

## Results

### P2R Inhibitors Protect Astrocytes in an Experimental NMOSD Mouse Model

**Fig. 1:**
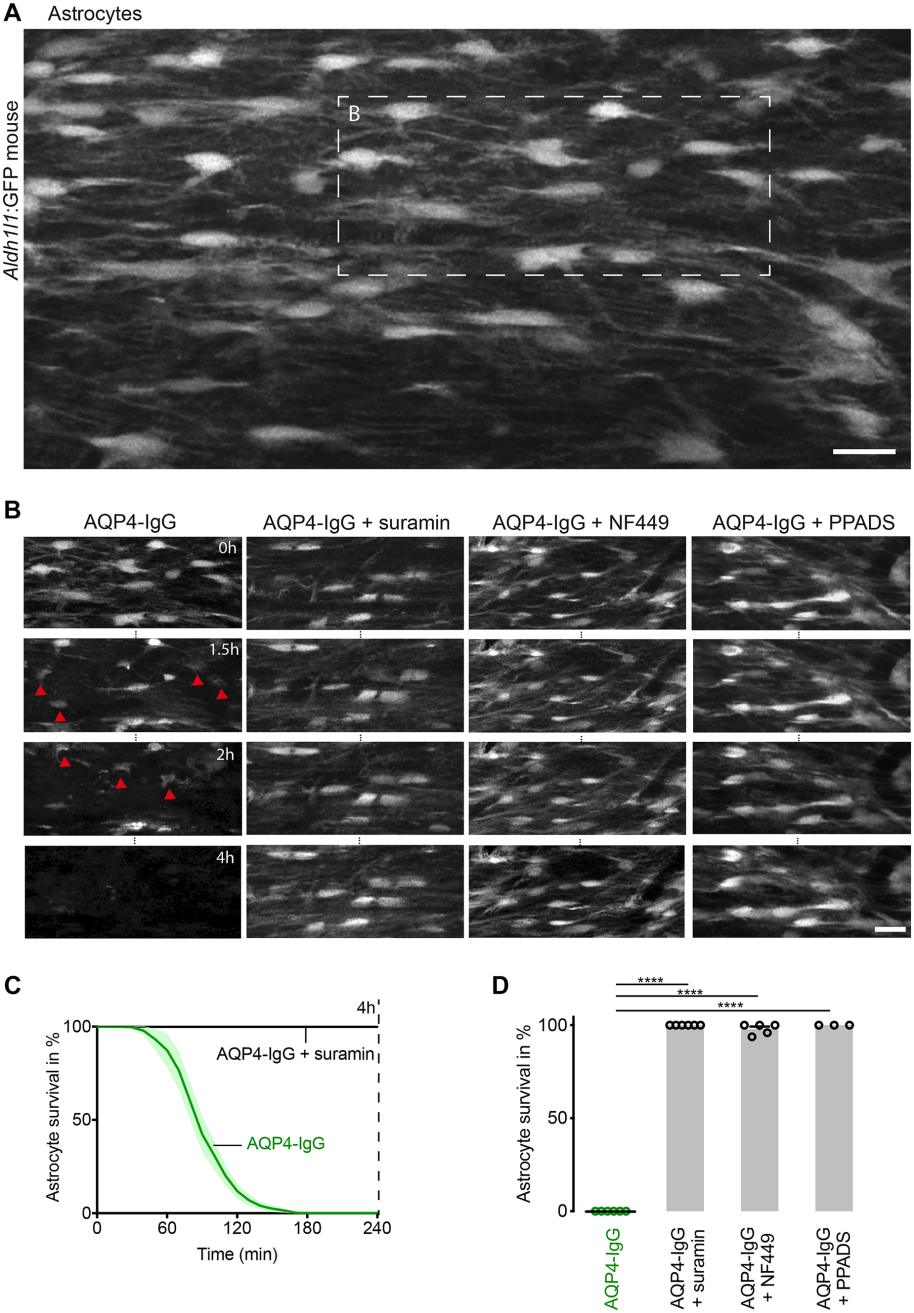
P2R inhibitors suramin, NF449, and PPADS protect astrocytes from AQP4-IgG-mediated complement-dependent cell death in vivo **A** Overview of the spinal cord imaging area in transgenic *Aldh1l1*:GFP mice visualized with in vivo two-photon microscopy. Scale bar 20 µm. Boxed area is time-lapsed in **B**, left. **B** In vivo time-lapse images of astrocytes over 4 h after the application of AQP4-IgG (150 µg/ml) and complement without (left) and with P2R blockers suramin, NF449, and PPADS or (right 3 series) to the spinal cord in vivo. Red arrowheads indicate dying astrocytes. Scale bars apply to all panels: 20 µm. **C** Percentage of surviving astrocytes in the spinal cord over time after application of AQP4-IgG with complement alone (*n* = 6) or together with suramin (1000 µM, *n* = 6). Dashed line illustrates the 4-h time point of the quantification shown in **D**. **D** Percentages of surviving astrocytes 4 h after AQP4-IgG/complement application alone or together with suramin (1000 µM, *n* = 6), NF449 (500 µM, *n* = 5) or PPADS (1000 µM, *n* = 3). ****p < 0.0001 compared to AQP4-IgG alone, Kruskal–Wallis test followed by Dunn’s multiple comparisons test. *n* indicates the number of mice. Data are presented as mean ± standard error of the mean

To explore the impact of P2R inhibitors on AQP4-IgG-mediated astrocytic pathology in vivo, we used an imaging-compatible NMOSD mouse model that we have previously described [32]. In brief, this model takes advantage of a two-photon imaging approach to visualize cellular structures in the dorsal spinal cord of anesthetized mice. Here, we used the transgenic mouse line *Aldh1l1:*GFP for astrocyte-specific GFP expression. Establishing a spinal cord imaging window of the dorsal column, allowed us to apply heat-inactivated AQP4-IgG-positive NMOSD samples derived from a NMOSD patient, together with a source of complement (pooled healthy sera, used at a concentration of 20% in all in vivo experiments), and to follow AQP4-IgG-mediated astrocyte toxicity under stable conditions over several hours. With this approach, application of AQP4-IgG and complement rapidly depleted GFP-labeled astrocytes (Fig. 1A–D and supplementary Movie S1, the mean of surviving astrocytes after 4 h in % ± SEM: 0.00 ± 0.00, *n* = 6). Surprisingly, in the presence of the P2R inhibitor suramin (1000 µM), all astrocytes survived (astrocyte survival after 4 h: 100.00 ± 0.00, *n* = 6: Fig. 1B–D). Similarly, two other widely used P2R inhibitors NF449 (500 µM) and PPADS (1000 µM) also efficiently protected astrocytes from AQP4-IgG-mediated death (astrocyte survival after 4 h, NF449: 97.93 ± 1.31, *n* = 5; PPADS: 100.00% ± 0.00%, *n* = 3: Fig. 1B and D).

### P2R Inhibitors Prevent Complement Deposition

**Fig. 2:**
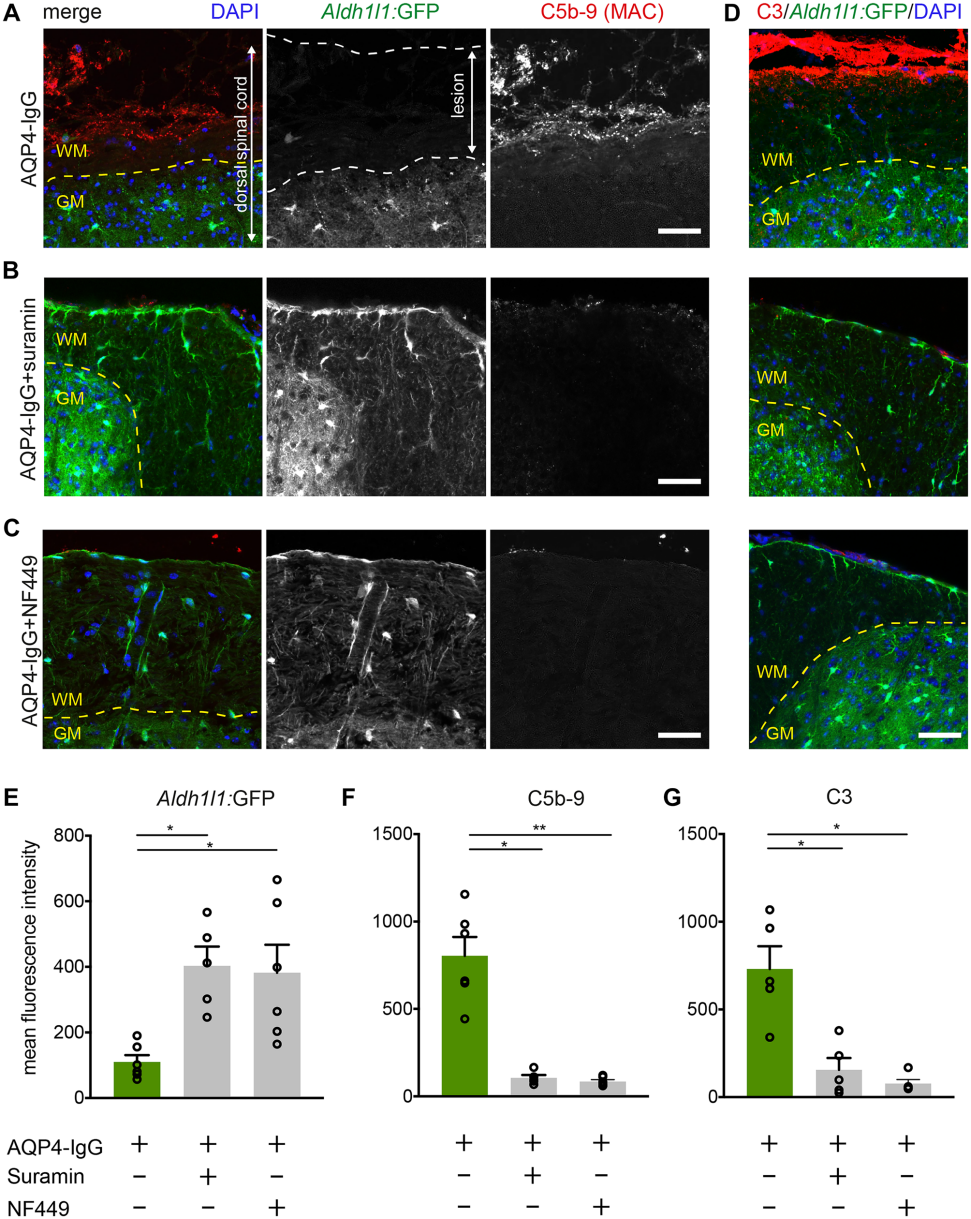
Treatment with P2R inhibitors prevents complement deposition in vivo. **A**–**C** Confocal images of spinal cord cryosections of *Aldh1l1:*GFP mice (astrocytes in green) treated with AQP4-IgG and complement for 4 h in vivo in the presence and absence of suramin (1000 µM) or NF449 (500 µM), stained for nuclei (DAPI, blue) and for C5b-9-complex (MAC, red). Note prominent complement deposition in animals treated with AQP4-IgG alone that was absent in animals additionally treated with suramin or NF449. **D** C3 deposition in AQP4-IgG-mediated lesions which is absent after treatment with P2R inhibitors. Scale bar 50 µm in A-D. Differences in mean fluorescence intensity are presented as aligned dot plot with mean ± standard error of the mean for GFP as a marker of surviving astrocytes (**E**), for C5b-9 staining (**F**) and for C3 staining (**G**). In **E**, **F**, AQP4-IgG: *n* = 6 animals, AQP4-IgG + suramin; *n* = 5 animals, AQP4-IgG + NF449: *n* = 6 animals. In **G**, AQP4-IgG: *n* = 5 animals, AQP4-IgG + suramin; *n* = 5 animals, AQP4-IgG + NF449: *n* = 4 animals. * < 0.05, ** < 0.01, Kruskal–Wallis test

Complement activation is a critical event in AQP4-mediated pathology, and its deposition is a hallmark of NMOSD lesions in humans and in NMOSD animal models [27, 45–47]. Therefore, we performed immunohistochemical stainings for deposition of the membrane attack complex (C5b-9), which is an important terminal step in the antibody-mediated classical complement pathway. Staining of fixed spinal cord sections after NMOSD lesion induction revealed a low astrocyte specific GFP signal in the dorsal spinal cord (Fig. 2A and D: mean fluorescence intensity for GFP ± SEM: 109.6 ± 21.3) in line with astrocytic death. Prominent MAC deposition was visible in the lesions in animals treated with AQP4-IgG and complement (Fig. 2A and F: C5b-9 ± SEM: 804.4 ± 107.6). In contrast, treatment with suramin (1000 µM) preserved GFP intensity signal from astrocytes in the dorsal spinal cord (Fig. 2B and E; GFP ± SEM: 403.1 ± 58.7) and led to a very low corresponding MAC signal (Fig. 2B and F: C5b-9 ± SEM: 105.8 ± 16.7). The treatment with the P2R inhibitor NF449 (500 µM) revealed comparable results (Fig. 2C, E and F: GFP ± SEM: 381.8 ± 85.6 and C5b-9 ± SEM: 85.6 ± 10.5).

Given that MAC deposition is the last step in the complement cascade, we aimed to test whether earlier steps could still be activated. As C3 deposition is a decisive step in the complement activation cascade, we stained tissue against human C3. Treatment with P2R inhibitors (suramin or NF449) significantly reduced C3 signal (Fig. 2D and G; 1000 µM suramin: C3 ± SEM: 156.2 ± 67.2 and 500 µM NF449: C3 ± SEM: 76.9 ± 23.0 compared to C3 ± SEM: 730.7 ± 129.9 in untreated NMOSD lesions). These results demonstrate that the treatment with P2R inhibitors protected astrocyte from death and prevented complement deposition in vivo.

### P2R Inhibitors Interfere with Human IgG Antibodies

**Fig. 3:**
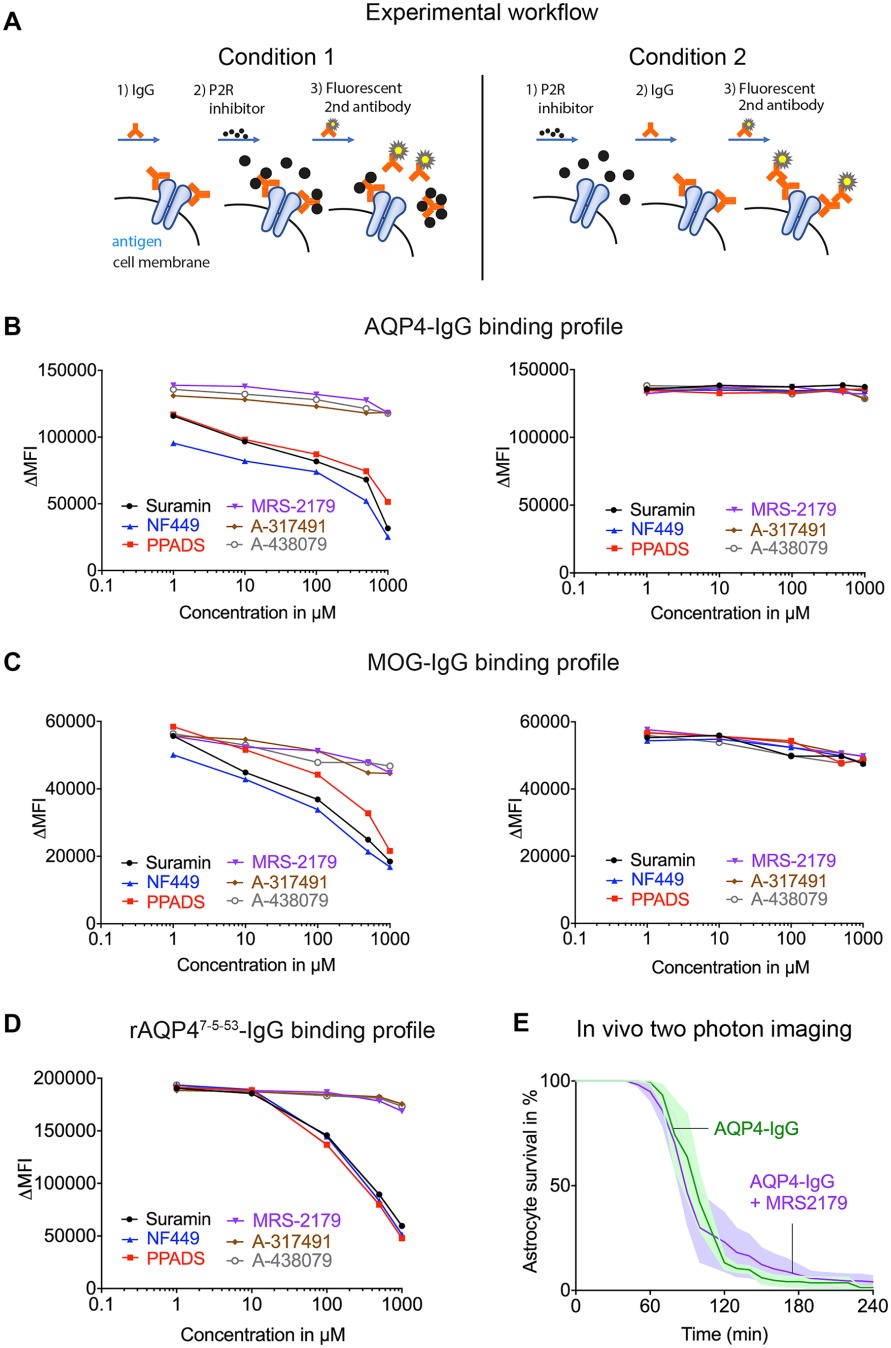
P2R inhibitors interfere with human IgG but do not block antigen binding. **A** Illustrative scheme of the experimental workflow for the cell-based binding experiments. Condition 1 (left): First, AQP4-IgG were applied to the AQP4 protein expressing LN18 cell line, followed by application of one of 6 different purinergic inhibitors and finally stained with fluorescently labeled secondary antibodies. Condition 2 (right): First, P2R inhibitors were applied to the AQP4 expressing cells followed by application of AQP4-IgG and later by staining with secondary antibodies (with washing steps in-between). ΔMFI was measured with FACS. **B** AQP4-IgG binding profile in the presence of each P2R inhibitor at different concentrations, respectively, under condition 1 on the left and condition 2 on the right. Application of P2R inhibitors showed a remarkable drop in ΔMFI with increasing concentrations under condition 1. Note that 3 purinergic inhibitors (A-317491, A-438079, and MRS-2179) had no effect under both conditions. **C** The same approach as in **B**, but with patient derived MOG-IgG. **D** The same as in **B** and **C**, but with rAQP4^7−5–53^-IgG. **E** Percentage of surviving astrocytes in vivo after application of AQP4-IgG alone (*n* = 3) and together with MRS-2179 (*n* = 4). MRS-2179 failed to protect astrocytes from death in the spinal cord NMOSD mouse model (*p* > 0.05), Mann–Whitney *U* test; *n* indicates the number of mice

To further analyze how P2R inhibitors interfere with NMO-IgG, we used a cell-based binding assay with two experimental conditions (illustration in Fig. 3A). Transfecting the LN18 cell line with human AQP4-antigen or MOG-antigen, we screened 6 commonly used P2R inhibitors (suramin, PPADS, NF449, MRS-2179, A-317491, A-438079) for their effects on AQP4- and MOG-IgG antibody binding analyzed by FACS. We chose these compounds because of their different structural properties and receptor selectivity. While suramin and PPADS are polycyclic aromatic compounds, representing non-selective P2R inhibitors, other P2R inhibitors such as MRS-2179, A-317491, and A-438079 show higher receptor selectivity. MRS-2179 has a high affinity to P2Y1 receptors [48], A-317491 to P2X3 and P2X2/3 receptors [49], and A-438079 is a selective P2X7R inhibitor [50]. Although NF449 has similar structure as suramin, it is considered to be a highly selective P2X1R inhibitor [51].

Under condition 1, (1) application of IgG antibodies was followed by (2) application of P2R inhibitors (concentrations 1 µM, 10 µM, 100 µM, 500 µM, 1000 µM) and (3) staining with the secondary fluorescently labeled antibodies (washing steps in between). Interestingly, the detection of binding of AQP4-IgG (100 µg/ml; derived from the serum of a NMOSD patient) or MOG-IgG (100 µg/ml, derived from the serum of a MOGAD patient) to AQP4- and MOG-expressing cells decreased in a dose-dependent manner in the presence of suramin, PPADS, or NF449 (Fig. 3B, C, left — Condition 1; representative graphs of ≥ 3 independent experiments). On the other hand, 3 P2R inhibitors (MRS-2179, A-317491, A-438079) did not affect antibody binding (Fig. 3B, C). In contrast, when (1) exposure to P2R-inhibitors was prior to (2) application of AQP4-IgG or MOG-IgG (condition 2, washing steps in between), the antibody-antigen binding was stable following the treatment of any of the P2R blockers (Fig. 3B, C) even in high concentrations (1000 µM). In a subset of experiments, we tested for C3b binding under condition 1 and observed a similar drop in fluorescence upon P2R exposure (data not shown). To rule out nonspecific effects when using human sera as antibody source, we tested P2R inhibitors together with the recombinant AQP4-IgG (rAQP4^7−5–53^, 5 µg/ml), which confirmed the same binding interference as observed with patient-derived NMOSD samples (Fig. 3D, representative graph of ≥ 3 independent experiments). The finding that some of the P2R inhibitors do not interfere with AQP4-IgG in cell-based assays prompted us to verify this observation in vivo (Fig. 3E). Two photon in vivo imaging of the spinal cord after application of AQP4-IgG and complement in the presence of MRS-2179 (1000 µM) led to prominent astrocytic death (Fig. 3E), with similar kinetics as observed in the control experiments without MRS-2179 treatment (mean of surviving astrocytes in % ± SEM: AQP4-IgG alone 1.35 ± 0.69, *n* = 3; AQP4-IgG + MRS-2179: 4.11 ± 3.10, *n* = 4). Together, these results suggest that disruption of complement activation in vivo is due to an interaction of P2R inhibitors with IgG-autoantibodies.

### SEC and CD Spectroscopy Reveal Partial Unfolding of IgG by P2R Inhibitors

**Fig. 4:**
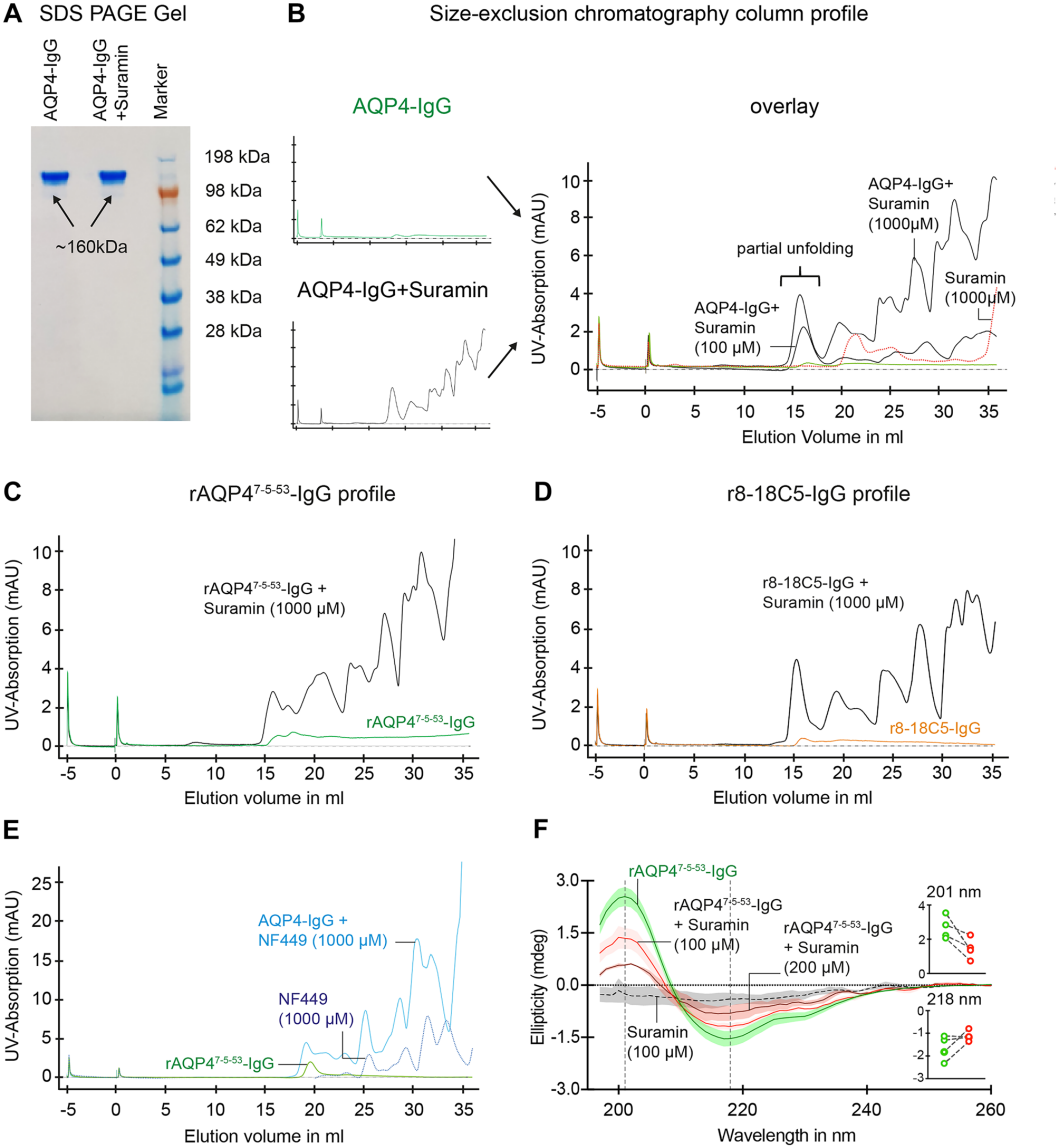
Size-exclusion chromatography and CD spectra of AQP4- and MOG-IgG with and without P2R inhibitors. **A** SDS-PAGE analysis of AQP4-IgG (50 µg) after purification and incubation without and with suramin (200 µM) for 2 h. **B** SEC profile of AQP4-IgG (50 µg) in native condition without suramin (green trace) and with 100 µM and 1000 µM suramin (black traces) as single traces and overlay. Suramin alone (1000 µM) is shown as a dashed red trace in the overlay. Note the partial unfolding of IgG in the presence of suramin. Absorption was measured at 280 nm as milli-Absorbance Units (mAU). **C**, **D** The same as in **B** for rAQP4^7−5–53^-IgG (left; green) and r818C5-IgG (right, orange). **E** SEC profile of AQP4-IgG (50 µg) in the absence (green) and presence of NF449 (blue, 1000 µM). NF449 alone (1000 µM) is depicted as a dashed trace (dark blue). Shown experiments are representative of 3 biological replicates. **F** Mean CD spectra of rAQP4^7−5–53^-IgG (0.15 mg/ml, green trace), of suramin alone (grey dashed trace), and of rAQP4^7−5–53^-IgG together with 100 µM suramin (red trace) or 200 µM suramin (dark red trace). Figure insets: signal changes (with 100 µM suramin) at 201 nm (top) and 218 nm (bottom), respectively. *n* = 4 samples

To further elucidate how P2R inhibitors might interact with AQP4-IgG, we first purified NMO-IgG (50 µg) and incubated with suramin (200 µM for 2 h). Analysis of AQP4-IgG on denaturing but non-reducing SDS-PAGE (Fig. 4A) showed no difference between native IgG and IgG treated with suramin. In both cases, immunoglobulin heavy and light chains were intact and attached to each other (AQP4-IgG: ~ 160 kDa; AQP4-IgG + suramin: ~ 160 kDa). However, more detailed analysis using SEC (Fig. 4B) revealed a striking profile change for AQP4-IgG treated with suramin, showing a partly unfolded protein pattern in contrast to the normal protein profile seen with untreated IgG (Fig. 4B). This effect was dose-dependent and could also be verified in the profile analysis of the rAQP4^7−5–53^-IgG (Fig. 4C). We wondered whether the observed unfolding by P2R inhibitors is specific for AQP4-IgGs or potentially applies also to other autoantibodies, such as MOG-IgG. Indeed, in native condition, the SEC of a recombinant anti-MOG-antibody r818C5 with suramin demonstrated unfolding pattern similar to AQP4-IgG (Fig. 4D). Performing SEC of AQP4-IgG in the presence of the P2R inhibitor NF449 showed comparable profile changes as observed with suramin (Fig. 4E). Altogether, the change in SEC pattern of AQP4- and MOG-IgG by P2R inhibitors is likely the reason for abolished antibody-mediated cellular damage observed in vivo.

To confirm that AQP4-IgG undergoes a conformational change upon exposure to P2R inhibitors, we decided to add experiments using far-UV circular dichroism spectroscopy (CD) as a complementary technique. Being a powerful and sensitive method to study changes in the conformation of a protein, CD spectroscopy allows to follow the folding and unfolding of proteins [52]. Measuring CD spectra of naïve rAQP4^7−5–53^-IgG demonstrated positive maxima at 201 nm, negative maxima at 218 nm, and zero ellipticity at around 208 nm (Fig. 4F), a typical signal expected from naïve IgGs [53, 54]. Suramin alone did not show any significant CD signal compared to background measurements. In contrast, when measuring rAQP4^7−5–53^-IgG together with 100 µM suramin, we detected a clear decrease in peak intensity at 201 nm (from 2.67 ± 0.33 to 1.46 ± 0.31, *p* < 0.05, *n* = 4 samples) and a trend towards an increase at 217 nm without any shift in wavelength (Fig. 4F). CD ellipticity signals changed in a dose-dependent manner. Altogether, these data further support a conformational change of IgGs upon P2R inhibitor exposure being responsible for the failure of AQP4-IgG-mediated immune response.

## Discussion

Purinergic antagonists have been used for the treatment of a broad range of inflammatory diseases [16] with varying outcome. Our results now add a protective effect of P2R inhibitors against NMO-IgG-mediated astrocyte death in vivo to this spectrum of immune-modulating activities. Our findings indicate that this effect is likely a direct interaction of commonly used P2R inhibitors with IgG-antibodies, resulting in partial unfolding of the antibodies and disruption of complement involvement, but likely not antigen binding. Thus, our study provides new insights into the immune-modulating mechanisms of P2R inhibitors and suggests considering their potential to acutely suppress antibody-mediated effects in autoimmune neurological conditions.

To be relevant to clinical settings, the doses needed to block IgG effects have to be attainable in vivo. Although the concentrations of purinergic antagonists used in our in vivo experiments were rather high and probably above clinically safe concentrations, our ex vivo data indicate that already lower dosages are enough to significantly interact with IgGs (e.g., 100 µM corresponding to 129.7 mg/l for suramin). This is in a similar range as clinically used suramin, which in its use to treat parasitic infections can reach plasma concentrations up to ~ 250 mg/l after a single administration [16, 55–57]. Another aspect that probably needs to be considered is the relationship between the number of inhibitor and target molecules which also seems to play a role for receptor-independent actions of P2R inhibitors such as suramin [22]. Needless to say, further studies are needed to estimate the potential of P2R inhibitors in neuroinflammatory disorders, also in view of the fact that in our experiments, these drugs were applied locally.

Nevertheless, the here presented interaction with antibodies might be a favorable option in certain clinical applications. For instance, during the acute onset phase of an NMOSD attack, where the blood–brain-barrier is anyway leaky and complement activation at astrocyte endfeet is believed to be an amplifying step [58, 59], direct inhibition of AQP4-IgG could be a beneficial add-on therapy, gaining time for more targeted complement-modifying interventions [60, 61]. Indeed, this might even allow the use of P2R inhibitors that are not believed to be CNS-penetrant, such as suramin or PPADS [16].

For further exploration, in addition to NMOSD, other autoimmune disorders involving strong complement activation could be a suitable context, such as Myasthenia gravis [62], bullous pemphigoid [63], or Goodpasture syndrome [64]. In fact, performing SEC on different IgG subtypes from different species (human IgG1, IgG2, IgG3, and IgG4 versus mouse IgG2a, IgG3) demonstrated that suramin led to a conformational change of all mentioned IgG subtypes (data not shown). These results suggest that P2R inhibitors do not only affect specific autoantibodies from IgG subtype.

Importantly, there are previous studies that have investigated individual P2R inhibitors in disease settings that involve complement-mediated damage, even though the mechanism of beneficial effects either remained obscure or was ascribed to P2R blockage. For instance, in other neurological conditions such as stroke [18, 19], traumatic brain and spinal cord injury [65–67], or amyotrophic lateral sclerosis [68], treatment with P2R inhibitors in corresponding rodent models has shown beneficial effects and favorable outcome during recovery phase [69, 70]. Outside neurological field, in age-related macular degeneration, it has been shown that topical treatment with PPADS prevents membrane-attack complex deposition in choroidal blood vessels and retinal pigment epithelium [71]. In $a$-hemolysin-mediated erythrocyte lysis [21], used as a model for sepsis, it has been found that PPADS and suramin inhibit complement-induced lysis of human erythrocytes, which was interpreted as blocking amplification by ATP release. Finally, in an in vitro autoimmune glomerular mesangial cell lysis model [72], it was found that suramin inhibits cell lysis. Although in these studies a direct effect of the P2R inhibitors on IgG effector function was not considered, these previous studies are compatible with our interpretation of a potentially broad effectiveness of P2R inhibitors in modifying complement-mediated pathology. Thus, also in the context of non-neurological autoimmunity, further exploration of P2R inhibitors as acute modulators of immune effector functions might be worthwhile.

However, our study comes with several limitations and caveats that need to be considered in this context. First, at the moment, the clinical use of P2R inhibitors is limited by substantial side effects, including renal and hepatic toxicity [16]. Thus, for further development in clinical context, it would be necessary to generate new P2R inhibitor derivatives with optimized pharmacological profiles while reducing off-target toxicity. Indeed, besides its action as an inhibitor of purinergic signaling, suramin was reported to have many different off-targets. Suramin is a large molecule carrying numerous negative charges at physiological pH and therefore very probable to bind to various proteins, such as enzymes [16, 22], Na + /K + -ATPase [73], and other receptors than purinoceptors, e.g., such as GABA receptors [74]. Second, given the serendipitous nature of the IgG interaction, P2R inhibitors may have additional diverse effects on immune receptors or effector proteins that we have not detected. One possibility that we cannot fully exclude is a direct inhibitory effect on complement components. Still, the dose dependency of the effect in the complement-free in vitro binding assays and the concordance of these findings with the in vivo observations can be sufficiently explained by IgG unfolding via P2R inhibitors.

Third, our study showed that not all P2R inhibitors had the same effects, with some inducing partial IgG unfolding and completely blocking complement-dependent cytotoxicity in vivo, while others are being inefficacious. We speculate that the difference in IgG interaction might relate to the degree of a P2R inhibitors’ amphiphilic properties that could make them act as “surfactant-like” proteins [75], but clearly, more systematic structural investigation and efforts of chemical modification are needed to provide a rational explanation of the observations we report.

In conclusion, we report here that P2R inhibitors are able to protect CNS cells from antibody-mediated autoimmune attacks by partial unfolding of AQP4- and MOG-IgG and thus disruption of complement activation. This mechanism is of relevance for the use of purinergic inhibitors in ex vivo and in vivo models of autoimmunity and may encourage research for novel and more efficient derivatives that can be used without safety concerns in humans.

## Supplementary Information

Below is the link to the electronic supplementary material.

Supplementary file1 (PDF 332 KB)

Supplementary file2 (PDF 321 KB)

Supplementary file3 (PDF 320 KB)

Supplementary file4 (PDF 317 KB)

Supplementary file5 (PDF 325 KB)

Supplementary file6 (AVI 1701 KB)

## Data Availability

Disclosure forms provided by the authors are available with the online version of this article.
